# Elevated levels of neutrophil related chemokine citrullinated histone H3, interleukin-8 and C-reaction protein in patients with immune checkpoint inhibitor therapy: predictive biomarkers for response to treatment

**DOI:** 10.1186/s12935-023-02994-8

**Published:** 2023-08-14

**Authors:** Xueping Wang, Hao Huang, Lin Zhang, Yaxian Wu, Yingsheng Wen, Xuezi Weng, Qi Chen, Wanli Liu

**Affiliations:** 1 Department of Laboratory Medicine, State Key Laboratory of Oncology in South China, Collaborative Innovation Center for Cancer Medicine, Guangdong Esophageal Cancer Institute, Cancer Center, Sun Yat-sen University, Guangzhou, 510060 China; 2 Department of Laboratory Medicine, The First Affiliated Hospital of Sun Yat-sen University, Guangzhou, 510060 China; 3 Department of Thoracic Surgery, State Key Laboratory of Oncology in South China, Collaborative Innovation Center for Cancer Medicine, Guangdong Esophageal Cancer Institute, Cancer Center, Sun Yat-sen University, Guangzhou, 510060 China; 4 Department of Blood Transfusion, State Key Laboratory of Oncology in South China, Collaborative Innovation Center for Cancer Medicine, Guangdong Esophageal Cancer Institute, Cancer Center, Sun Yat-sen University, Guangzhou, 510060 China

**Keywords:** Cancer, H3Cit, Neutrophil extracellular traps, Immune checkpoint inhibitors, Serum biomarker

## Abstract

**Background:**

Immune checkpoint inhibitor (ICI) therapy has been used in various tumors. The biomarkers predictive of a response to ICI treatment remain unclear, and additional and combined biomarkers are urgently needed. Secreted factors related to the tumor microenvironment (TME) have been evaluated to identify novel noninvasive predictive biomarkers.

**Methods:**

We analyzed 85 patients undergoing ICI therapy as the primary cohort. The associations between ICI response and all biomarkers were evaluated. A prediction model and a nomogram were developed and validated based on the above factors.

**Results:**

Seventy-seven patients were enrolled in the validation cohort. In the primary cohort, the baseline serum levels of H3Cit, IL-8 and CRP were significantly higher in nonresponder patients. A model based on these three factors was developed, and the “risk score” of an ICI response was calculated with the formula: “risk score” = 3.4591×H3Cit + 2.5808×IL8 + 2.0045 ×CRP– 11.3844. The cutoff point of the “risk score” was 0.528, and patients with a “risk score” lower than 0.528 were more likely to benefit from ICI treatment (AUC: 0.937, 95% CI: 0.886–0.988, with sensitivity 80.60%, specificity 91.40%). The AUC was 0.719 (95% CI: 0.600-0.837, P = 0.001), with a sensitivity of 70.00% and specificity of 65.20% in the validation cohort.

**Conclusions:**

A model incorporating H3Cit, IL-8 and CRP has an excellent prediction ability for ICI response; thus, patients with a lower “risk score” selectively benefit from ICI treatment, which may have significant clinical implications for the early detection of an ICI response.

## Background

Cancer immunotherapy, especially immune checkpoint inhibitors (ICIs), has revolutionized systemic treatments for advanced tumors, including melanoma, non-small cell lung cancer (NSCLC), squamous cell carcinoma of the head and neck (HNSCC), hepatocellular carcinoma (HCC), biliary tract cancer and gastric carcinoma (GC). Programmed cell death 1 (PD-1) is one of the inhibitory immune checkpoints expressed on T cells, B cells, NK cells, and some myeloid cells. Currently, ICIs targeting the PD-1/PD-L1 pathway are the most common treatment, such as the anti-PD-1 antibodies nivolumab and pembrolizumab and the anti-PD-L1 antibody atezolizumab [1–3]. Patients accepting ICI therapy have a better survival time and an unprecedented higher cure rate than those treated with conventional therapies, such as chemotherapy, radiation therapy and targeted therapy. However, only a limited number of patients (~ 20%) benefit from immunotherapy, and some cancers that initially respond to immunotherapy may ultimately relapse [4].

Expression of the PD-L1 protein, the presence of tissue infiltrating lymphocytes (TIL), tumor mutational burden (TMB) measured in tumor tissue or peripheral blood, Eastern Cooperative Oncology Group (ECOG) performance status, routine laboratory parameters such as lactate dehydrogenase and peripheral blood cell counts have been proposed to predict clinical benefits from ICI treatment [5–7]. However, because of the cost and complex methodologies they require for an accurate assessment, these biomarkers cannot be routinely used in the clinic [8]. Therefore, it is imperative to identify additional specific and sensitive biomarkers to identify patients who are likely to respond to treatment with ICIs. Recent studies have suggested that biomarker combination approaches may be the future of response prediction to ICI therapies rather than single biomarkers; however, there are few reports about these biomarkers.

The interaction between tumor cells and the immune system in the tumor microenvironment (TME) is involved in the initial malignant transformation of normal cells to tumor growth and progression, which also plays a crucial role in determining the outcome of the host antitumor response. Cytokines are released in response to cellular stress, injury, or infection and they stimulate the restoration of tissue homeostasis to restrict tumor development and progression. However, persistent cytokine secretion in the setting of unresolved inflammation can promote tumor cell growth, inhibit apoptosis, and drive tumor cell invasion and metastasis [9]. IL-8 is a member of the CXC glutamic acid-leucine-arginine motif bearing (ELR+) family, which is secreted not only by cancer cells but also by myeloid cells and fibroblasts infiltrating tumors in the TME. IL-8 is a frequently upregulated chemokine in human malignant tissues, and a fraction of patients show increased circulating IL-8 levels in advanced stages [10–13]. Neutrophil extracellular traps (NETs) are decondensed chromatin fibers attached to granular enzymes that are released from activated neutrophils. NETs have already been found to be involved in a variety of disease processes as well as tumors, and they play a key role in both proliferation and malignant transformation [14, 15]. IL-8 has emerged as a potent biomarker to predict ICI responses in patients, and its upstream role in neutrophil modulation and its pleiotropic pro-tumor effects make IL-8 and its receptors suitable therapeutic targets [16]. IL-8 has been shown to be upregulated on tumor-infiltrating lymphocytes (TILs), suggesting that blocking IL-8 could enhance antitumor immunity [17], which is also related to tumor-associated neutrophils [18]. The value of NETs as biomarkers has yet to be fully characterized, and technical issues need to be resolved. Citrullinated histone H3 (H3Cit) has been proposed as a target biomarker reflecting the level of NET. The clinical investigation of TME-related biomarkers could also lead to novel insights into ICI therapy.

In recent years, clinical and laboratory risk factors for predicting the response to ICIs in cancer patients have been identified. In this retrospective cohort study, we hypothesized that biomarkers reflecting the TME could be used to monitor the response to immunotherapy. In this study, the levels of H3Cit, IL-8 and other TME biomarkers were assessed in a panel of patients treated with PD-1/PD-L1 inhibitors.

## Methods

### Study design and population

We performed a retrospective cohort analysis of 85 patients who received anti-PD1/PD-L1 immunotherapy at Sun Yat-sen University Cancer Center from October 1, 2019, to September 31, 2020. All patients met the following inclusion criteria: (1) patients over 18 years of age; (2) patients received ICI therapy (pembrolizumab, nivolumab, durvalumab or atezolizumab); and (3) baseline assessments were performed with a computed tomography scan (CT scan) of the chest and abdomen within 2 weeks before treatment, and then the oncological outcomes were assessed every 2 cycles of treatment. The independent validation cohort included 77 consecutive patients with the same inclusion and exclusion criteria as those in the primary cohort from October 1, 2020, to May 31, 2021, at Sun Yat-sen University Cancer Center.

**Fig. 1:**
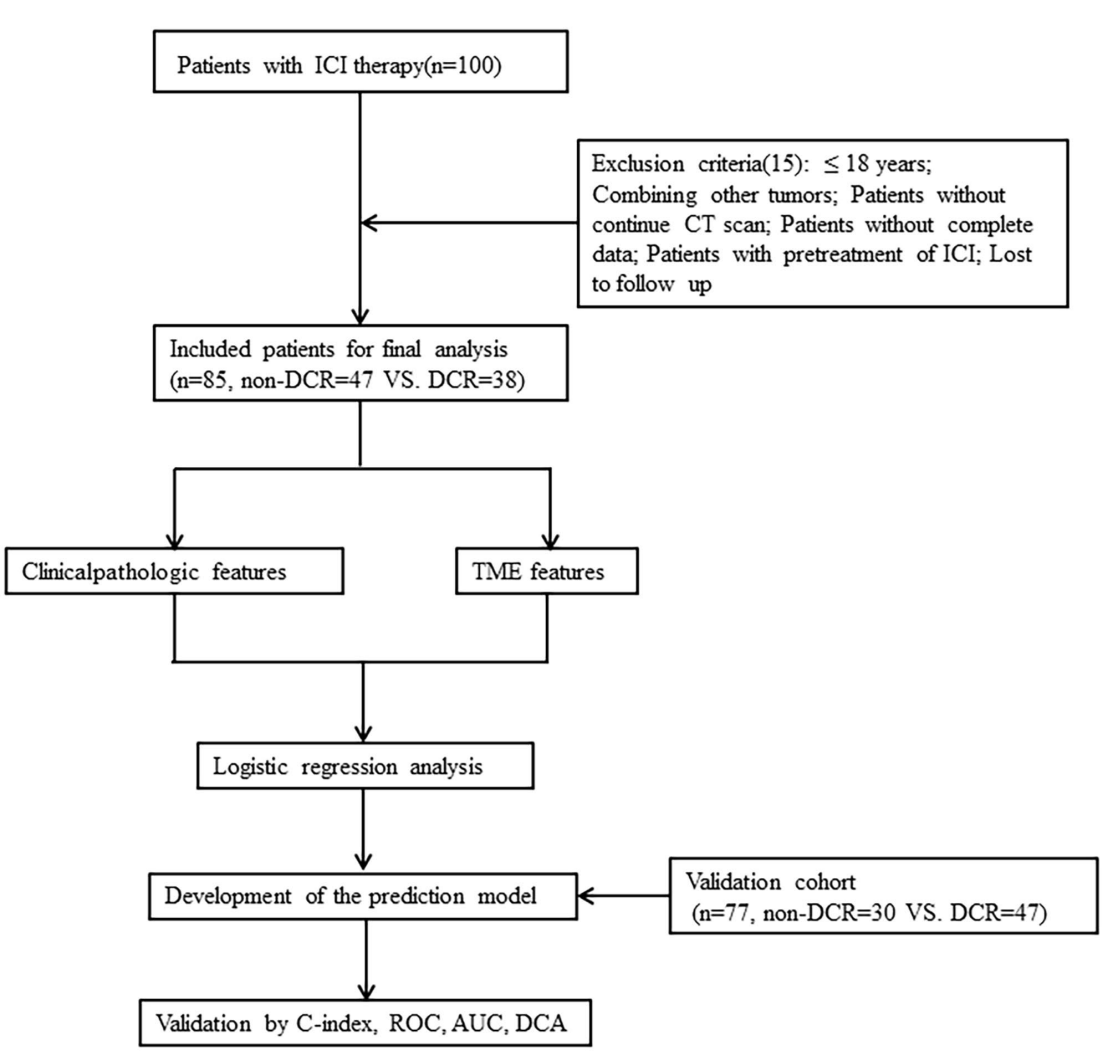
Flowchart of our study

The study design for the identification of a predictive signature for patients with cancer treated with ICIs is displayed in Fig. 1.

### Treatments

The following ICI treatments were included: pembrolizumab, nivolumab, durvalumab and atezolizumab. All ICIs were selected by the treating physicians according to the current guidelines and clinical standards. Dosing of the ICI was performed according to the European Medicines Agency marketing authorizations. Prior therapy was defined as prior systemic treatments.

### Endpoints and assessments

Blood samples were collected in a serum separation tube at room temperature on the morning of the ICI treatment. All blood samples were drawn by venipuncture and clotted at room temperature within 30 min. Serum samples were obtained by centrifuging at 3500 r/min and 4 °C for 10 min. After centrifugation, the precipitate was discarded, and the supernatant serum was stored at − 80 °C until use. All samples were processed within one hour. Whole blood samples were processed within one hour. Peripheral whole blood markers were measured before each administration of the ICI. Patients who were treated with ICI were followed up using computed tomography approximately every 2 to 3 months and they were classified into 3 groups based on their treatment response according to the Response Evaluation Criteria in Solid Tumors version 1.1: (1) Partial response (PR): total reduction of the diameter of the target lesions (enhanced arterial phase) by ≥ 30%; (2) Stable disease (SD): the diameter of the target lesion was not reduced to that in PR and not increased to that in progressive disease; (3) Progressive disease (PD): the diameter of the target lesion increased by at least 20% compared with the baseline value or the appearance of new lesions.

Furthermore, the ICI efficacy was also evaluated by a durable clinical response (DCR; PR/SD that lasted for more than 6 months) or a non-DCR [19]. Overall survival (OS) and progression-free survival (PFS) were calculated starting with the first administration of the ICI. OS was defined as the interval between the initiation of ICI therapy and the time of death from any cause. PFS was estimated from the time of ICI therapy to the date of disease progression or death due to any cause.

### Laboratory analysis

The concentration of H3cit in the serum was measured using an ELISA kit developed by Cayman Chemicals (Ann Arbor, MI, USA). This assay employs a monoclonal antibody specific for histone H3 citrullinated at R2, R8, and R17 (clone 11D3). The lower limit of detection of this assay is 0.1 ng/mL, and the upper limit is 31 ng/mL.

Immunohistochemistry (IHC) was used to detect the expression of H3Cit. Briefly, the antigen was retrieved by microwave-heating the tissue in a 10 mM sodium citrate buffer at pH = 6.0. These sections were then blocked for 20 min with a blocking solution (0.1% Triton-X, 10% normal goat serum in 1× PBS) at room temperature (RT). After that, the antibodies H3Cit (1:200, ab18521) were added to these tissue samples, respectively, and placed overnight at 4 °C. The samples were then incubated using biotin-labelled secondary antibodies at RT for 30 min. The HRP-labelled SP working medium was added and incubated at RT for 30 min.

Serum levels of soluble IL-8, IL-18 and IL-18BP were assayed using ELISA kits (CUSABIO, China). Their assay ranges are 31.25–2000 pg/mL, 31.25–2000 pg/mL and 15.6–1000 pg/mL, with detection limits of 7.110 pg/mL, 7.8 pg/mL and 3.9 pg/mL, respectively. For all of these tests, the intra-assay precision was less than 8%, and the interassay precision was less than 10%. All serum levels of biomarkers were assayed in duplicate according to the manufacturer’s instructions.

The level of protein, as well as the complete blood cell and platelet counts, were assayed by routine laboratory techniques.

### Statistical analysis

SPSS 16.0 (IBM, Chicago, IL, USA) and R software (version 3.1.4; http://www.Rproject.org) were used for the statistical analysis. Baseline clinical characteristics of the patients were summarized as absolute frequencies (percentages) and assessed with the chi-square test. Continuous variables were reported as medians [25th–75th percentile] and compared by the use of the Mann–Whitney U test. Cutoff values of serum protein levels were estimated with a receiver operating characteristic (ROC) curve. The Mann–Whitney U test was used to compare the median OS and PFS. The Kaplan–Meier method was used to estimate OS and PFS, which were compared using the log-rank test. Univariate and multivariate regression analyses were used to analyze the risk factors to predict the ICI response. For multivariate analysis, variables with a known and/or strong univariate association with the ICI response were selected. Risk factors based on multivariate logistic analysis were applied to develop a diagnostic model for patients with ICI therapy. To quantify the discrimination performance of the nomogram, Harrell’s C-index was evaluated. In brief, a C-index value greater than 0.75 is considered to represent relatively good discrimination. Calibration was performed by observing the survival probability with Kaplan–Meier estimation. The decision and ROC curves were plotted for the model and the other biomarkers. A p value of < 0.05 was considered significant.

## Results

### Baseline clinicopathological characteristics

The distribution of the patients’ demographics and baseline clinical characteristics were well balanced between the two cohorts. In the primary cohort (Table 1), the median age was 48 years; 64.70% were men. The tumor entities were non-small cell lung cancer (NSCLC, 12 patients, 14.10%), nasopharyngeal carcinoma (NPC, 37 patients, 43.50%), gastrointestinal tumors (GC, 22 patients, 25.90%), and melanoma (MM, 14 patients, 16.50%), and 71 patients (83.50%) received prior therapy. A total of 69.90% (58) of patients had an ECOG performance status of 1 or more. There were 47 (55.29%) non-DCR patients. In the validation cohort, 66.20% were men. NSCLC (21 patients, 27.30%), NPC (24 patients, 31.20%), GC (14 patients, 28.57%) and melanoma (18 patients, 23.40%) were included in this study. Prior therapy was used in 62 patients (80.50%). More than 59.70% (46) of patients had an ECOG performance status of 1 or more. There were 30 patients classified in the non-DCR group.

**Table 1:** Patient demographics and baseline clinical characteristics in the primary and validation cohorts

Characteristic	Primary cohort (85) No. (%)			Validation cohort (77) No. (%)			P Value
	All patients(85)	non-DCR (47)	DCR (38)	All patients(77)	non-DCR (30)	DCR (47)	
Age, years, n (%)							
≥ 48	45(52.9%)	31(66.0%)	14(36.8%)	51(66.2%)	18(60.0%)	33(70.2%)	0.086
< 48	40(47.1%)	16(34.0%)	24(63.2%)	26(33.8%)	12(40.0%)	14(29.8%)	
Sex, n (%)							
Male	55(64.7%)	30(63.8%)	25(65.8%)	51(66.2%)	18(60.0%)	33(70.2%)	0.838
Female	30(35.3%)	17(36.2%)	13(34.2%)	26(33.8%)	12(40.0%)	14(29.8%)	
ECOG score, n (%)							
0	25(30.1%)	13(27.7%)	12(31.6%)	31(40.3%)	9(30.0%)	22(46.8%)	0.179
1	58(69.9%)	34(72.3%)	26(68.4%)	46(59.7%)	21(70.0%)	25(53.2%)	
Smoke, n (%)							
Yes	29(34.1%)	19(40.4%)	10(26.3%)	21(27.3%)	10(33.3%)	11(23.4%)	0.346
No	56(65.9%)	28(59.6%)	28(73.7%)	56(72.7%)	20(66.7%)	36(76.6%)	
Alcohol, n (%)							
Yes	13(15.3%)	7(14.9%)	6(15.8%)	15(19.5%)	8(26.7%)	7(14.9%)	0.482
No	72(84.7%)	40(85.1%)	32(84.2%)	62(80.5%)	22(73.3%)	40(85.1%)	
Tumor classification, n (%)							
Lung cancer	12(14.1%)	9(9.1%)	3(7.9%)	21(27.3%)	7(23.3%)	14(29.8%)	0.068
Nasopharyngeal carcinoma	37(43.5%)	20(42.6%)	17(47.7%)	24(31.2%)	4(13.3%)	20(42.6%)	
Gastrointestinal tumors	22(25.9%)	13(27.7%)	9(23.7%)	14(18.2%)	7(23.3%)	7(14.9%)	
Melanoma	14(16.5%)	5(10.6%)	9(23.7%)	18(23.4%)	12(40.0%)	6(12.8%)	
Stage, n (%)							
III	33(38.8%)	16(34.0%)	17(44.7%)	24(31.2%)	5(16.7%)	19(40.4%)	0.308
IV	52(61.2%)	31(66.0%)	21(55.3%)	53(68.8%)	25(83.3%)	28(59.6%)	
Distant metastasis sites, n (%)							
Lung	18(23.7%)	10(22.7%)	8(25.0%)	19(27.1%)	13(34.2%)	6(18.8%)	0.230
Liver	17(22.4%)	9(20.5%)	8(25.0%)	9(12.9%)	2(5.3%)	7(21.9%)	
Bone	19(25.0%)	12(27.3%)	7(21.9%)	15(21.4%)	10(26.3%)	5(15.6%)	
Other	22(28.9%)	13(29.5%)	9(28.1%)	27(38.6%)	13(34.2%)	14(43.8%)	
No. of metastatic sites, n (%)							
< 2	64(75.3%)	33(70.2%)	31(81.6%)	52(67.5%)	16(53.3%)	37(76.6%)	0.274
≥ 2	21(4.7%)	14(29.8%)	7(18.4%)	25(32.5%)	14(46.7%)	11(23.4%)	
Prior therapy, n (%)							
Yes	71(83.5%)	40(85.1%)	31(81.6%)	62(80.5%)	23(23.3%)	39(83.0%)	0.618
No	14(16.5%)	7(14.9%)	7(18.4%)	15(19.5%)	7(76.7%)	8(17.0%)	

### Analysis of TME biomarkers in hematological samples

The serum was collected before patients receiving ICI treatment. The concentrations of serum H3Cit (21.27 vs. 7.65, P < 0.001), IL-8 (289.81 vs. 91.02, P < 0.001), CRP (24.01 vs. 1.40, P = 0.022) and SAA (57.35 vs. 7.05, P < 0.001) were significantly higher in the non-DCR patients than the DCR group, while the concentration of serum ALB (31.00 vs. 28.81, P = 0.030) was lower in the non-DCR than the DCR group (Table 2). There were no significant differences in any other baseline clinical variables between the two groups.

**Table 2:** Peripheral blood laboratory inflammatory biomarkers

Inflammatory biomarkers	All patients (85, median/IQR)	non-DCR (47, median/IQR)	DCR (38, median/IQR)	*P* Value
H3Cit (ng/mL)	11.54(5.76–28.22)	21.27(9.45–65.46)	7.65(2.52–11.29)	*P* < 0.001
IL-8 (pg/mL)	118.64(48.03-319.98)	289.81(125.94-670.42)	91.02(45.63-181.91)	*P* < 0.001
IL-18 (pg/mL)	221.38(159.98-382.02)	245.88(191.38–489.00)	210.94(158.53-323.98)	*P* = 0.258
IL-18BP (pg/mL)	947.59(459.64-1316.98)	766.93(459.64-1294.38)	1100.72(250.78-1797.94)	*P* = 0.528
WBC (10^9^/L)	6.19(5.32–7.90)	6.47(5.44–8.32)	5.69(4.83–6.95)	*P* = 0.090
NEU (10^9^/L)	4.04(3.09–5.36)	4.29(3.29–5.57)	3.94(2.91–5.17)	*P* = 0.356
LYM (10^9^/L)	1.51(1.04–1.79)	1.55(1.06–1.98)	1.41(1.02–1.76)	*P* = 0.331
MO (10^9^/L)	0.41(0.33–0.55)	0.46(0.36–0.58)	0.38(0.29–0.52)	*P* = 0.074
EO (10^9^/L)	0.10(0.06–0.17)	0.10(0.06–0.17)	0.10(0.05–0.16)	*P* = 0.487
BASO (10^9^/L)	0.03(0.02–0.05)	0.03(0.02–0.05)	0.03(0.02–0.05)	*P* = 0.531
PLT (10^9^/L)	273.00(212.50–318.00)	273.00(201.00-310.00)	279.50(213.25–327.50)	*P* = 0.714
NLR	2.95(2.19–4.45)	2.92(2.06–4.52)	3.03(2.39–3.87)	*P* = 0.939
LMR	3.00(2.08–4.64)	2.85(2.00-5.07)	3.17(2.35–4.33)	*P* = 0.838
PLR	203.29(138.44-256.74)	203.03(115.48-255.14)	205.31(165.10-258.71)	*P* = 0.466
ALB (g/L)	42.10(39.25-46.00)	41.30(38.00-43.70)	43.95(40.28–46.63)	*P* = 0.020
GLB (g/L)	30.61(26.19–33.44)	30.94(28.07–35.48)	29.44(25.27–33.07)	*P* = 0.163
CRP (mg/L)	9.00(1.44–30.34)	17.31(4.09–78.19)	2.58(0.97–10.17)	*P* < 0.001
SAA (mg/L)	13.99(5.15-102.35)	51.60(8.85-220.95)	7.70(4.15–38.80)	*P* = 0.001

### Associations between the inflammatory biomarker concentrations and the ICI response

**Fig. 2:**
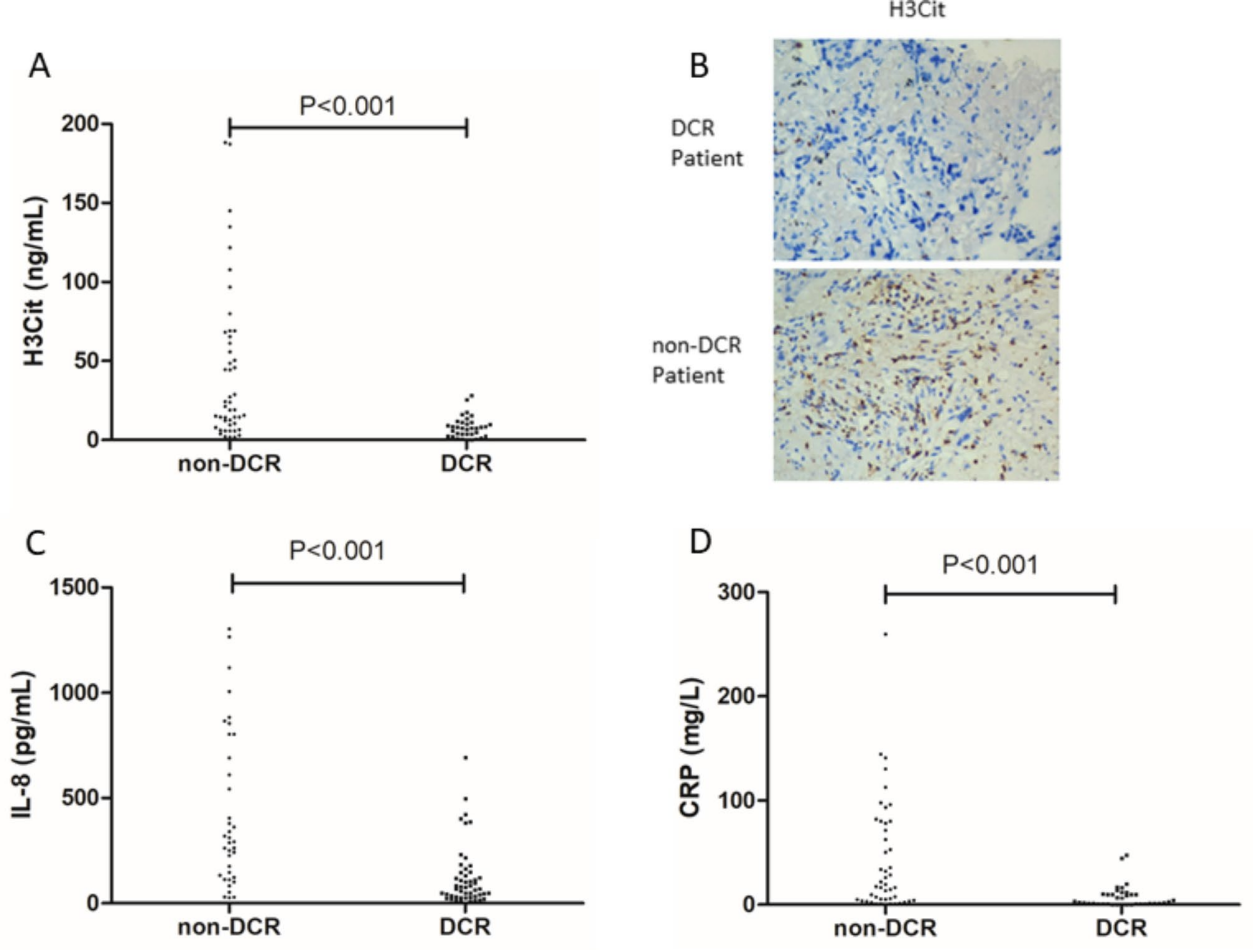
Baseline levels of H3Cit, IL-8 and CRP between ICI responder and nonresponder patients. (**A**) The difference in the serum levels of H3Cit in the primary cohort (P < 0.001). (**B**) IHC of H3Cit expression between ICI responder and nonresponder in lung cancer. (**C**) The difference in the levels of IL-8 in the primary cohort (P < 0.001). (**D**) The difference in the serum levels of CRP in the primary cohort (P < 0.001)

**Fig. 3:**
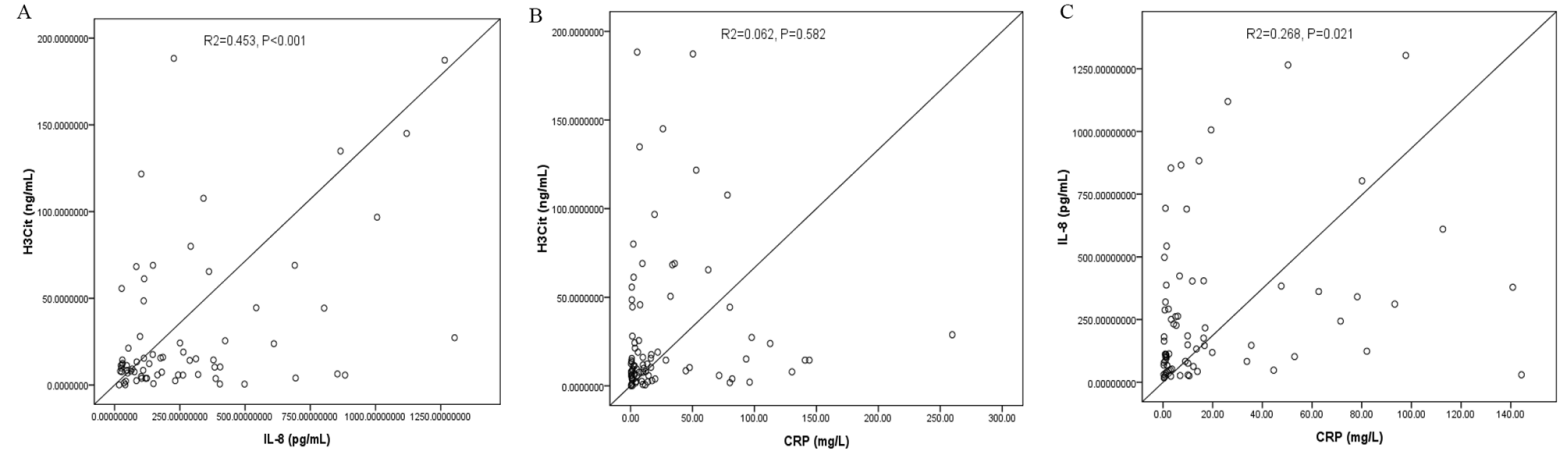
Correlations between the concentrations of serum H3cit, IL-8 and CRP. (**A**) The H3Cit level was positively correlated with the level of IL-8 (Spearman R^2^ = 0.453, *P* < 0.001); (**B**) The H3Cit level was not correlated with the CRP level (Spearman R^2^ = 0.062, *P* = 0.582); (**C**) The IL-8 level was correlated with the CRP level (Spearman R^2^ = 0.268, *P* = 0.021)

To explore the predictive value of ICI treatment, we evaluated the association between the TME biomarker levels and the clinical benefits in patients treated with ICIs. In the primary cohort, univariate and multivariate Cox proportional hazard regression analyses were used to estimate the relationship between all of the biomarkers and the ICI response. Univariate analysis suggested that 6 variables were significantly associated with the response to ICI: age (HR, 3.1.046; 95% CI, 1.007–1.086, P = 0.020), H3Cit (HR, 11.393; 95% CI, 3.874–33.503, P < 0.001), IL-8 (HR, 8.857; 95%, 3.027–25.918, P < 0.001), WBC (HR, 2.912; 95% CI, 1.191–7.121, P = 0.019), MO (HR, 2.462; 95% CI, 1.009–6.009, P = 0.048), ALB (HR, 0.222; 95% CI, 0.087–0.563, P = 0.002), CRP (HR, 8.910; 95% CI, 2.954–26.877, P < 0.001), and SAA (HR, 5.558; 95% CI, 2.041–15.137, P = 0.001). Among these, we performed multivariate logistic regression analysis. The multivariate analysis showed that baseline H3Cit (HR, 31.343; 95% CI, 4.871-201.663, P < 0.001), IL-8 (HR, 14.116; 95% CI, 2.071–96.207, P = 0.007), and CRP (HR, 11.751; 95% CI, 1.542–89.539, P = 0.017) were significantly associated with ICI efficacy (Table 3; Fig. 2A-C). Furthermore, we used IHC to detect H3Cit expression in lung cancer tissues treated with ICI treatment, higher expression of H3Cit was were present on non-DCR patient than DCR patient (Fig. 2B). Clinical factors, such as stage, ECOG score, and sites of metastases, were not associated with a response. Furthermore, the serum H3cit level remained positively associated with the level of IL-8 (R^2^ = 0.453, P < 0.001) (Fig. 3A-C).

**Table 3:** Association between H3Cit, IL-18, CRP, and ICI response (univariable and multivariable competing-risk regression models)

Characteristics	Univariate analysis		Multivariate analysis	
	HR (95% CI)	P value	HR (95% CI)	P value
Age, year <48/≥48	1.046(1.007–1.086)	0.020		
Sex				
Male/Female	0.846(0.422–1.693)	0.636		
ECOG score				
0/1	1.066(0.413–2.751)	0.896		
Smoking				
Yes/No	0.482(0.191–1.216)	0.122		
Alcohol				
Yes/No	0.914(0.287–2.907)	0.879		
Tumor classification				
Lung cancer/Nasopharyngeal carcinoma/Gastrointestinal tumors/Melanoma	0.673(0.418–1.083)	0.103		
Stage				
III/IV	1.568(0.651–3.778)	0.316		
Distant metastasis sites				
Lung	1.667(0.286–9.708)	0.570		
Liver	0.799(0.273–2.336)	0.682		
Bone	2.571(0.534–12.378)	0.239		
Other	1.733(0.403–7.462)	0.460		
No. of metastatic sites				
<2/≥2	0.602(0.227–1.594)	0.307		
Combination therapy				
Yes/No	1.524(0.497–4.667)	0.461		
H3Cit (ng/mL)				
<12.11/≥12.11	11.393(3.874–33.503)	0.000	31.343(4.871-201.663)	0.000
IL-8 (pg/mL)				
<221.88/≥221.88	8.857(3.027–25.918)	0.000	14.116 (2.071–96.207)	0.007
IL-18 (pg/mL)				
<201.48/≥201.48	1.000(1.000–1.000)	0.486		
IL-18BP (pg/mL)				
< 98.78/≥98.78	1.000(0.998–1.001)	0.596		
WBC (10^9^/L)				
<5.80/≥5.80	2.912(1.191–7.121)	0.019		
NEU (10^9^/L)				
<4.44/≥4.44	2.076(0.851–5.064)	0.108		
LYM (10^9^/L)				
<1.94/≥1.94	1.371(0.617–3.047)	0.439		
MO (10^9^/L)				
<0.44/≥0.44	2.462 (1.009–6.009)	0.048		
EO (10^9^/L)				
<0.06/≥0.06	1.395E9(0.000-)	0.999		
PLT (10^9^/L)				
<235.0/≥235.0	1.391(0.569–3.402)	0.469		
NLR				
<3.98/≥3.98	1.935(0.722–5.188)	0.189		
LMR				
<4.74/≥4.74	1.879(0.670–5.269)	0.231		
PLR				
<223.58/≥223.58	1.605(0.655–3.930)	0.713		
ALB (g/L)				
<41.95/≥41.95	0.222(0.087–0.563)	0.002		
GLB (g/L)				
<38.23/≥38.23	0.588(0.122–2.837)	0.508		
CRP (mg/L)				
<14.19/≥14.19	8.910(2.954–26.877)	0.000	11.751(1.542–89.539)	0.017
SAA (mg/L)				
<12.95/≥12.95	5.558(2.041–15.137)	0.001		

### Development and discrimination of the prediction model

**Fig. 4:**
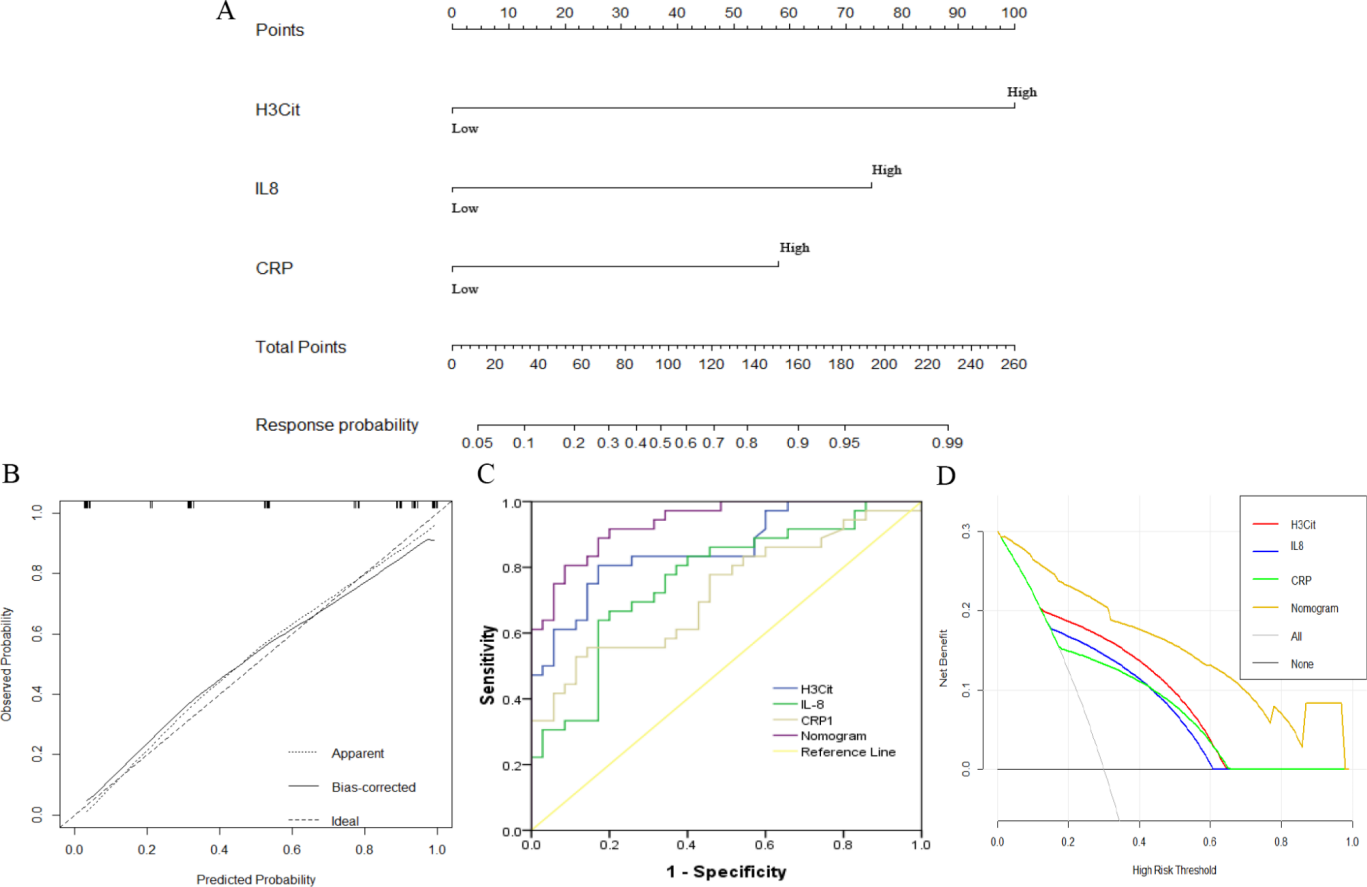
Development and validation of the prediction nomogram in the primary cohort. (**A**) Nomogram to predict the response in patients with ICI therapy. The nomogram is valued to obtain the probability of the ICI response by adding up the points identified on the points scale for each variable, which included the level of H3Cit, IL-8 and CRP. (**B**) Calibration curve of our nomogram. (**C**) The AUC of our nomogram was 0.937 (95% CI: 0.886–0.988, P < 0.001). (**D**) The results of the decision curve analysis. Decision curve analysis for the nomogram and other previously reported variables. The gray line represents the assumption that all patients are within the responder group. The thin black line represents the assumption that all patients are within the nonresponder group

A novel model was constructed to predict the ICI response based on the above inflammatory biomarkers identified by Cox analysis. Patients with high levels of H3Cit (P = 0.033), IL-8 (0.027) and CRP (P = 0.030) were more likely to be considered ICI nonresponders (Fig. 4A). According to the regression coefficients of the model, the “risk score” of an ICI response was calculated by the formula: “risk score” = 3.4591×H3Cit + 2.5808×IL8 + 2.0045 ×CRP– 11.3844. The value of the formula: H3Cit (categorical variable): ≥12.11: 2/<12.11: 1, IL8 (categorical variable): ≥221.88: 2/<221.88: 1; CRP (categorical variable): ≥14.18: 2/<14.18: 1. The cutoff point of “risk score” was 0.528; thus, patients could be divided into two groups by “risk score” as follows: patients with “risk score” ≥0.528 were classified as ICI non-DCR patients, while patients with “risk score” <0.528 were classified into ICI DCR groups. Discrimination was performed by using a concordance index (C-index). Calibration was evaluated by comparing the means of the predicted survival with the observed Kaplan–Meier survival, with the x-axes representing actual survival estimated by the nomogram and the y-axes representing observed survival calculated by the Kaplan–Meier method. The C-index for ICI response was 0.937. The AUC (ROC curve) of this model was 0.937 (95% CI: 0.886–0.988, with sensitivity 80.06%, specificity 91.40%), which showed an optimal prediction for an ICI response (Fig. 4B-C). The calibration curve of the model demonstrated good agreement in predicting an ICI response between prediction and observation in the primary cohort. In the validation cohort, the C-index for response prediction was up to 0.719. The AUC was 0.719 (95% CI: 0.600-0.837, P = 0.001), with a sensitivity of 70.00%, specificity of 65.20%, PPV of 66.79%, and NPV of 68.49%.

### Clinical use

**Fig. 5:**
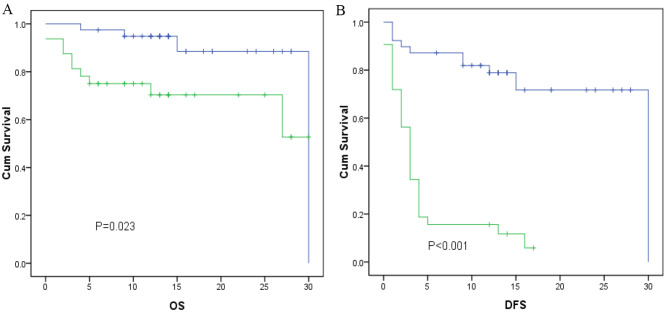
Kaplan–Meier survival curves of the nomogram in the primary cohort. (**A**) Kaplan–Meier survival curves for OS; (**B**) Kaplan–Meier survival curves for DFS

The decision curve analysis of the model for ICI response is presented in Fig. 4D, which shows that if the threshold probability of a patient is > 10%, the model is beneficial in predicting an ICI response to patients receiving ICI treatment. According to the nomogram in this range, the net benefit was comparable. The nomogram for predicting an ICI response was more advantageous than the biomarker only (IL-8, H3Cit, CRP) in predicting an ICI response. Based on the nomogram we developed in this study, the patients were subdivided into a low-risk group and a high-risk group, which showed good classification for patients in the primary cohort. Additionally, the OS between the 2 groups was 11.91 vs. 15.79 (P = 0.023) months, and the DFS between the 2 groups was 4.18 vs. 13.82 (P < 0.001) months (Fig. 5A-B).

## Discussion

Immunotherapy targeting immune checkpoint pathways is effective in many cancers, such as melanoma, NSCLC, NPC and GC. The blockade of PD-1/PD-L1 is the standard treatment for numerous malignancies [20]. However, clinical benefits vary across tumor types, and most patients progress despite treatment. Early identification of patients who are insensitive to treatment could avoid ineffective therapies with potentially serious adverse effects. In the present study, we explored the serum proteins associated with the ICI response in various tumors. A multivariate analysis validated that the levels of H3Cit, IL-8 and CRP across treatment settings were significantly associated with the ICI response across four tumor types. Then, we developed a model and “Risk score” formula using relatively large datasets from the primary cohort. Additionally, an increase in H3Cit was associated with the level of IL-8. We also proposed a clinically meaningful cutoff “risk score” of 0.528, which indicates that patients with a higher score (“risk score”≥ 0.528) do not benefit from ICI therapy. The constructed model is an easy-to-use, preoperative, and individualized parameter that can be easily measured in conventional blood specimens in clinical settings for predicting an ICI response.

H3Cit has been established as a marker for neutrophil extracellular traps (NETs), as citrullination of histone H3 by PAD4 leads to chromatin decondensation and subsequent NET formation. NETs are webs of DNA coated with specific proteins such as histones, MPO, cathepsin G, leukocyte proteinase 3 (PR3), and neutrophil elastase (NE) [21]. NETs have been related to the progression of several tumors, such as lung adenocarcinoma, large B-cell lymphoma, breast cancer, and ovarian cancer [22, 23]. Importantly, in many of these diseases, NETs preferentially appear in advanced stages and, at least in breast and colon cancer, seem more prominent in the liver metastases of these patients. According to most of these studies, the abundance of NETs correlates with a worse prognosis and disseminated disease. Yang L et al. showed that NETs promote cancer metastasis via NET-DNA contact with CCDC25 in both breast cancer and CRC [24]. Xiao Y et al. reported that NETs promote breast cancer lung metastasis through cathepsin C [25]. However, few studies have focused on the role of NETs in primary tumor growth. Nie M et al. [23] showed that NETs promote tumor growth and dissemination based on TLR9 agonistic activity exerted on lymphoma cells, and Demers M et al. [26] described that primary tumors grow slower in PAD4-deficient mice with LLC lung adenocarcinomas.

Furthermore, the ability of NETs to impair immunotherapy has been unclear. The possible mechanism is: DNAse I produced in the liver by AAV vectors reduced the presence of NETs in colon cancer metastases and enhanced local CD8 + T-cell infiltration [27]; orthotopic tumors using Kras-induced pancreatic cancer cells (KPC cells) engrafted in Pad4-deficient mice showed increased infiltration of activated CD8 + T cells and were more sensitive to PD-1 blocking mAbs [28]; and interleukin-17 has played a key role in the production of NETs via the induction of CXCR1/2 agonist chemokines, which attract neutrophils and elicit NETs.

IL-8, also known as CXCL8, is a proinflammatory CXC chemokine. Malignant tumor cells secrete IL-8 under certain environmental stresses, including hypoxia and chemotherapy agents [29]. IL-8 signals are mediated through interactions with the G protein-coupled receptors CXCR1 or CXCR2, which activate conjugated G proteins and then activate PLC, AC, PLD, PI3K, JAK2, ras and other signaling molecules. The level of IL-8 is increased in a variety of malignant tumor cells and is closely related to the proliferation, migration, invasion, angiogenesis and epithelial mesenchymal transformation of tumor cells [30, 31]. Tumor immune escape is one of the main characteristics in the process of tumor cell generation and metastasis. IL-8 has been shown to play an important role in tumor immune escape by inducing PD-L1, inhibiting apoptosis of tumor cells, promoting the EMT process in tumor cells, promoting angiogenesis in the tumor microenvironment, and recruiting immunosuppressive cells [32, 33]. Tumor-produced IL-8 tends to increase neutrophils or myeloid-derived suppressor cells (MDSCs) and leads to the induction of an immunosuppressive TME [34]. In a previous study, Alfaro C et al. [22, 35, 36] discovered that IL-8 can induce the formation of NETs by neutrophils and thereby entrap cancer cells ex vivo by adhesive mechanisms in coculture.

IL8 has been reported to favor cancer progression and metastases via different mechanisms, including pro-angiogenesis and the maintenance of cancer stem cells, but its ability to attract and functionally modulate neutrophils and macrophages is arguably one of the most important factors. IL8 not only recruits neutrophils to tumor lesions but also triggers the extrusion of NETs. The relevance and mechanisms underlying the contribution of both neutrophils and NETs to cancer development and progression are starting to be uncovered and they include both direct effects on cancer cells and changes in the tumor microenvironment, such as facilitating metastasis, awakening micrometastases from dormancy, and facilitating escape from cytotoxic immune cells [35, 37].

H3Cit, a biomarker of NETs, predicts the risk of mortality in patients with cancer [38, 39], and elevated baseline serum IL-8 and CRP levels were associated with adverse outcomes in melanoma, NSCLC, and RCC [40]. Although the contribution of a possible negative predictive effect of NET, IL-8 and CRP cannot be fully resolved due to the retrospective design and statistical considerations of this study, patients with high pretreatment NET, IL-8 and CRP levels are less likely to benefit from immune checkpoint inhibitors. To date, biomarkers such as microsatellite instability, TMB and PD-L1 have shown some defects in predicting ICI response. The development of a model based on serum H3Cit, IL-8 and CRP could expand the biomarker arsenal for optimal ICI treatment use in tumors that currently lack clinically useful biomarkers. The performance of the model was similar in both the primary and validation cohorts, indicating that the nomogram model had a strong predictive ability. In addition, this model had better performance than any single biomarker. Thus, this comprehensive and personalized risk score calculation model might be useful for stratification. We determined that 0.528 is a clinically relevant stratification cutoff risk score by using pooled analyses for ICI response in several tumors, which indicates that patients with a higher score (risk score ≥ 0.528) do not benefit from ICI therapy.

There are limitations of the present study. The first is that this study is an exploratory study, and the size of the study cohort is small. We are now recruiting patients with several kinds of tumors in the primary and validation cohorts in our hospital. Moreover, we plan to apply this model in a prospective multicenter study of patients treated with PD-1/PD-L1 antibodies. The second is that the follow-up period where clinical data are available is relatively short, and we need to evaluate the significance of these serum markers in terms of the long-term clinical benefit and the relationship between our model and the prognosis. The third is that some studies report that NETs or IL-8 inhibitors could improve the effect of immunotherapy [41, 42], but the mechanism is unclear, which is one of our future research directions.

## Conclusion

we propose an easy-to-use, preoperative model based on TME biomarkers to predict the ICI response for individual patients. Our findings indicate that patients with a higher relative risk score, indicated by a “risk score” ≥0.528, do not benefit from ICI therapy. This approach has great application potential in clinical practice in terms of planning individual treatments. In a future study, we will further explore the mechanism of NETs in ICI treatment and hope to find combination drugs to improve the efficacy of ICIs.

## Data Availability

The authenticity of this article has been validated by uploading the key raw data into the Research Data Deposit public platform (www.researchdata.org.cn), number RDDB2022319883.

## Abbreviations

DCR: Durable clinical response
ECOG: Eastern Cooperative Oncology Group
H3Cit: Citrullinated histone H3
IL-8: Interleukin 8
IL-18: Interleukin 18
IL-18BP: Interleukin 18BP
WBC: White blood cell count
NEU: Neutrophil
LYM: Lymphocyte
MO: Monocyte
EO: Eosinophils
BASO: Basophils
PLT: Platelet
NLR: Neutrophil/lymphocyte ratio
LMR: Lymphocyte/monocyte ratio
PLR: Platelet/lymphocyte ratio
ALB: Albumin
GLB: Globulin
CRP: C reactive protein
SAA: Serum amyloid A
